# Prevalence and Risk Factors Associated with Gross Pulmonary Lesions in Slaughtered Pigs in Smallholder and Commercial Farms in Two Provinces in the Philippines

**DOI:** 10.3389/fvets.2018.00007

**Published:** 2018-02-13

**Authors:** John I. Alawneh, Christopher R. Parke, Eduardo J. Lapuz, Jose E. David, Voltaire G. Basinang, Augusto S. Baluyut, Tamsin S. Barnes, Edwin C. Villar, Minnie L. Lopez, Joanne Meers, Patrick J. Blackall

**Affiliations:** ^1^The University of Queensland, School of Veterinary Science, Brisbane, QLD, Australia; ^2^Department of Agriculture Region 3, RADDL, Pampanga, Philippines; ^3^Provincial Veterinary Office of Bulacan, Malolos, Philippines; ^4^Provincial Veterinary Office of Pampanga, San Fernando, Philippines; ^5^The University of Queensland, Queensland Alliance for Agriculture and Food Innovation, Brisbane, QLD, Australia; ^6^Livestock Research Division PCAARRD-DOST, Los Baños, Philippines; ^7^Animal Health Division, Bureau of Animal Industry, Department of Agriculture, Quezon, Philippines

**Keywords:** lung scores, pigs, Philippines, respiratory disease, slaughter

## Abstract

A cross-sectional study of lungs from 1,887 randomly selected pigs from 471 farms from two provinces in the Philippines was carried out to estimate the prevalence of gross pathological lesions, identify potential risk factors and spatial clustering associated with high lung or pleurisy score farms. Lungs from pigs were scored at slaughter. Interviews with the farm managers were conducted to collect information about farm management and biosecurity practices. Of lungs examined, 48% had a lung score above 6 (maximum was 55) and 22% showed pleurisy. When data were aggregated at the farm level, commercial farms were at higher risk of being high lung score farms and high pleurisy farms compared to smallholder farms (*P* < 0.01). Variables that were associated with an increased risk of a farm being a high lung score farm included the presence of a market pen on the farm, not vaccinating against hog cholera and the presence of another piggery within 500 m. Practicing “feedback” (feeding pig manure, viscera or aborted material to pigs), presence of another piggery within 500 m, and allowing commercial livestock vehicles on farm were all associated with an increased risk of being a high pleurisy farm. Spatial analyses revealed a primary 9.6 km-radius cluster of 39 farms with high lung and pleurisy scores in the southeast of Bulacan province. High lung and pleurisy score farms could be targeted to improve on-farm disease control programs to reduce the risk of respiratory diseases. Clusters of high scoring farms could be prioritized for further investigations or for coordinating intervention efforts.

## Introduction

Clinical observations and slaughterhouse surveys have shown that the respiratory disease complex (RDC) is still a major concern for pig producers worldwide (1, 2), adversely affecting growth rates and increase morbidity and mortality among affected herds (3, 4).

Scoring pig lungs for gross pathological lesions at slaughter is a non-invasive tool to monitor herd health, allowing the recognition of chronic lung lesions and active respiratory disease occurring late in the pig production cycle (5). Catarrhal bronchopneuomnia and pleurisy, affecting the cranioventral lobes and the pleural membrane of the pleural sac, respectively, are the most frequent lung lesions encountered at slaughter (6, 7). *Mycoplasma hyopneumoniae* commonly is involved in cranioventral pneumonia and is often complicated by the presence of other bacteria such as *Actinobacillus pleuropneumoniae* and *Pasteurella multocida*, or viruses such as swine influenza virus or porcine reproductive and respiratory syndrome virus (5). The distribution of the pneumonic lesions may be localized or more generalized depending on the pathogens involved (5). An effective reduction in the risk of respiratory disease in the herd requires insights into the causative agents of RDC that are likely to be present in the herd (5, 8). For example, the United Kingdom adopted a national surveillance scheme to record gross pathology consistent with major respiratory pathogens such as *M. hyopneumoniae* and *A. pleuropneumoniae* (i.e., consolidation and pleurisy) at routine meat inspections (9). The results are recorded in a database and information is relayed back to herd-owners quarterly to alert them of likely infection status and to encourage implementation of disease control measures on affected farms.

In 2014, the Philippines pig industry produced just over 25 million pigs that yielded over 1.5 million metric tonnes of meat (10). A description of the Philippines pig industry has been described elsewhere (11). The Philippines is gradually shifting toward a more intensive pig production to meet local and international demand (12). Currently, there is limited information on respiratory diseases and the potential risk factors that could impact on the health and productivity of pigs in the Philippines. The aims of this study were to (a) estimate the prevalence of gross pathological lesions in lungs from slaughter-age pigs using lung scores; (b) identify and quantify potential risk factors associated with farms with high lung and pleurisy scores; and (c) investigate the spatial clustering of farms with high lung and pleurisy score.

## Materials and Methods

### Study Design

A cross-sectional study was conducted between October 2011 and March 2012 in two major pig-producing provinces (Bulacan and Pampanga) in Region 3 (Central Luzon) of the Philippines. The region was selected because it is one of the most intensive pig rearing regions in the country (13) and has reported major losses from acute respiratory disease in outbreaks that occurred in 2007–2009 (14).

The target population was finisher pigs in Bulacan and Pampanga provinces. The source population was finisher pigs that presented for slaughter during the study duration at cooperating slaughterhouses in the Bulacan and Pampanga provinces (*n* = 29; total number of abattoirs in the region was 32, 3 did not agree to participate). The study population was finisher pigs presented for slaughter where the farm of origin was located in Bulacan or Pampanga provinces and selected by the specified sampling strategy. To be able to identify a risk factor with an odds ratio (OR) of three or greater, assuming a statistical power of 80%, a level of significance of 95%, and 80% of unexposed farm have pigs with lung lesions (based on the findings of slaughterhouse lung score training conducted in the period between March and July 2011), and a prevalence of the risk factor in the population of 10%, 750 farms were required [(15); Winpepi, version 11.15]. To account for logistical difficulties and potential loss of farm traceability, 175 herds (19% of the desired sample size) were further added to the desired sample size.

### Data Collection

The farm enrollment process and farm questionnaire have been described elsewhere (11). Briefly, a cross-section of finisher pigs lungs were scored over a 25-week period between 1st October 2011 and 1st March 2012 in Bulacan and Pampanga provinces. In total, 110 intensive lung scoring session-days (time from start to finish of lung scoring in each slaughter day) were conducted. One hundred and ten days were selected to achieve our target sample size. Lung scoring days and slaughterhouses were randomly allocated (one slaughterhouse per day; on average, 5 days for lung scoring and 2 days team recovery in each week throughout the study period) using multistage stratified random sampling process as follows: sampling dates and slaughterhouses were stratified by province, and simple random sampling was used to allocate approximately 50% of slaughterhouse inspections to each province. A systematic random sampling process was used to select the sampling units (i.e., individual finisher pigs for lung scoring).

Field staff of the Provincial Veterinary Offices asked slaughterhouse meat inspectors and livestock traders in Bulacan and Pampanga provinces to record the following information prior to arrival for slaughter: (1) planned date of slaughter; (2) livestock trader name, address, and contact details; (3) livestock transporter name, address, and contact details; (4) source farm details; (5) date of purchase of pigs; and (6) number of pigs bought and intended for slaughter.

The following method was employed to identify pigs during slaughter for each intensive lung scoring sessions. A lung scoring team comprising three investigators was assembled, namely, the tagger-pacemaker, main lung scorer, and main lung scorer assistant. After lung scoring team decided on the sample start-up pig using a systematic random pig selection process, tagger-pacemaker then “tagged” the start-up and each of the subsequent pigs to be sampled (tagger action; tags secured to the pig’s gluteus muscle or the lung) with a green flag. These flags consisted of a bamboo stick, 30 cm long and 3 mm in diameter, with a 5 cm × 5 cm flap of green masking tape (3M™ Scotch^®^ performance masking tape 2308, 3M™ Ltd.). Pigs that were not included in the study sample were tagged with a black flag. The tagging order was determined by the order in which lungs was ready for scoring. Once scoring of a green tagged pig was completed, the MLS replaced the green tag with a black one.

The lung scoring system used in this study was based on the methodology by Straw et al. (16). The degree of consolidation in each lung lobe was assessed by estimating the percentage of the volume of each lobe of the lung with visual and palpable signs of consolidation. When the lung score was more than zero (maximum possible score is 55), these lesions were classified as either acute or chronic. Pleurisy was recorded as a score of zero—no pleurisy, one—fibrous adhesions between the lung lobes, two—pleurisy lesions over the caudal and or cranioventral lobes and also pleuritic lesions on the rib cage, or three—the lungs severely adhered to the rib cage and difficult to remove without tearing the lung tissue. Presence or absence of lesions on the diaphragmatic (caudal) lobes consistent with *A. pleuropneumoniae* (termed APP-Like) was recorded as present or absent. Pericarditis was recorded as present or absent. One experienced veterinarian was the lung scorer for all sessions.

Information provided by the traders were verified independently by trained surveyor teams (*n* = 2) conducting face-to-face interviews with the traders (at the time of slaughter) and the declared source farms. Discrepancies between trader and farm information were investigated and corrected where possible.

### Data Management

Records were excluded from this is study if there was inaccuracy in details supplied by livestock trader, or farms were located in other provinces, untraceable, or refused to participate. Moreover, a farm was also excluded if the date of slaughter was 6 weeks after pigs were sold to a trader. A 6-week interval was selected based on the fact that respiratory lesions (particularly *M. hyopneumoniae* infection) of pig lungs can resolve 8–12 weeks after lesions develop (17, 18).

A pig with a lung score greater than the median of all pigs was classified as having a high lung score and a pig with a pleurisy score of at least one was classified as having a high pleurisy score. Pig data were then aggregated at the farm level. A farm was classified as being a high lung score, pleurisy score, high APP-Like lesion, or high pericarditis farm if at least 50% of pigs from the farm were high lung or pleurisy score pigs, or were classified as having APP-Like lesions or pericarditis, respectively. When a farm had only one pig, the farm was classified based on the lesion status of that pig. Heat production units (HPUs) were calculated using the formula HPU = (0.17 × capacity of the finisher barn) + (0.17 × capacity of the nursery barn) + (0.45 × sow inventory) (19). HPU served as a proxy for herd size.

### Statistical Analyses

Descriptive statistics were determined for the lung and pleurisy scores of the pigs included in the study. These were compared to the descriptive statistics for the pig lung and pleurisy scores from the farms excluded from the analysis using Wilcoxon rank sum tests. The prevalence of farms with a high lung and pleurisy scores was calculated as the proportion of high lung and pleurisy score farms. The 95% confidence interval was estimated using robust SEs.

The production system was classified as either commercial or smallholder in accordance with definition used by the Philippine Bureau of Animal Statistics (13). The associations between candidate explanatory variables and two possible outcomes, farms being either high lung or pleurisy score farms, were assessed using univariable mixed-effects logistic regression models with abattoir fitted as a random effect. A *P* value ≤0.20 from a likelihood ratio test was used as a criterion for entry of an explanatory variable into the multivariable mixed-effects logistic regression model for that outcome. Potential instability in the multivariable modeling caused by high collinearity between explanatory variables was avoided by selecting only the most plausible variable from a correlated set (*r* ≥ 0.60) based on considerations including *a priori* importance of the risk factors, strength of associations, and missing values (20). Correlation between potential predictor variables was investigated using Pearson’s χ^2^ tests. The HPU of each farm was forced in the final models as a categorical variable to control for the potential confounding effect of farm size. Statistical interactions (only first order and biologically plausible interactions were considered) in the final multivariable model were tested and retained in the model if they were statistically significant. The overall fit of the final model was evaluated using the Hosmer and Lemeshow goodness of fit test implemented within the influence. ME package in R (21). Because ORs may lead to an overestimation of the size of the main effects (20), crude and adjusted ORs were converted to crude or adjusted risk ratios (RRs) using the technique by Beaudeau and Fourichon (22). Significance was declared at an alpha level of 0.05.

### Spatial Analyses

A farm was classified as being at high risk of respiratory disease if it was classified as a high lung score farm and/or a high pleurisy score farm. The number of high-risk farms per square kilometer was geographically interpolated using kernel smoothing techniques (23). The analyses were conducted using the spastat package (24) in R. The bandwidth parameter for the kernel function was fixed at 4 km and was calculated using normal optimal method (23). The locations of the smallholder and commercial farms included in the study were also plotted as points over this density map.

Spatial clustering of high-risk farms was assessed using the Spatial Scan Test performed in SaTScan™ version 9.1.1 software (Information Management Services Inc., Calverton, MD, USA), based on a purely spatial Bernoulli distributional assumption model and scanning for circular clusters with a maximal population threshold of 50% of sites. In this process, time remained dormant and the observed number of high-risk farms in a cluster was compared to the distribution of expected number under the assumption of independence. The statistical significance of clusters was determined through 9,999 permutations. Significant clusters were declared at an alpha ≤ 0.05.

## Results

A total of 2,489 pigs from 601 farms were enrolled during the study period (Table 1). Data from 602 pigs from 130 farms were excluded because the farms were untraceable, were located in another province, or the farmer/farm manager was unwilling to participate (Table 1). Lung scores from farms where the farmer/farm manager was unwilling or were located in a difference province were higher than scores from study farms (Table 1; *P* < 0.01). No farm was excluded because the 6-week period from purchase to slaughter was exceeded. Records from 1,887 pigs originating from 471 pig farms were included in the final analyses. Descriptive statistics of lung scores are shown in Table 2. Among all lungs examined, the median lung score was 6 and 48% of pigs were classified as high lung score, 51% had acute lung lesions, 28% had chronic lung lesions, 21% had no lung lesion, and 22% had pleurisy. The candidate explanatory variables assessed in univariable models are shown in Table 3. The prevalence of farms with a high lung (Bulacan 32%, 95% CI 25–38%; Pampanga 34%, 95% CI 28–40%; *P* = 0.19) or pleurisy score (Bulacan 16%, 95% CI 11–22%; Pampanga 12%, 95% CI 8–16%; *P* = 0.12) did not differ between provinces (Table 3). Variables related to sow feeding and vaccination practice were excluded from the analysis due to collinearity with grower-finisher feeding and vaccination practice.

**Table 1:** Comparison between lung scores and pleurisy scores recorded in Bulacan and Pampanga provinces between October 2011 and March 2012 for pigs from farms included and excluded from the analyses.

Farm classification	Number of farms	Number of lungs	Lung scores		Pleurisy scores^a^
			Median (Q1, Q3)	Min, Max	Median (Q1, Q3)
Study farms	471	1,887	6 (1, 16)	0, 55	0 (0, 1)
Farms where farmer was unwilling to participate^†^	29	71	8 (3, 19)	0, 54	0 (0, 2)
Untraceable farms	75	296	6 (2, 18)	0, 55	0 (0, 0)
Farms from other provinces^‡^	26	235	9 (4, 17)	0, 53	0 (0, 1)

*Q1, 25th percentile; Q3, 75th percentile*.

*^a^In all cases, the minimum was 0 and the maximum was 3*.

*^†,‡^The distribution of lung scores from farms where the farmer was unwilling to participate and farms from other provinces were different to the distribution of lung scores from study farms (Wilcoxon’s signed rank test *P* < 0.01)*.

**Table 2:** Number of pig farms, number of lungs examined, and descriptive statistics of lung and pleurisy scores stratified by province and farm production system recorded between October 2011 and March 2012.

Province	Production system	Number of farms	Total number lungs	Range (number of lungs/farm)	Lung scores		Pleurisy scores^a^
					Median (Q1, Q3)	Min, Max	Median (Q1, Q3)
Bulacan	Smallholder	162	491	1–70	4 (0, 13)	0, 53	0 (0, 0)
	Commercial	44	309	1–52	10 (4, 20)	0, 54	0 (0, 2)
	Total	206	800	1–70	6 (1, 16)	0, 54	0 (0, 1)
Pampanga	Smallholder	209	441	1–37	2 (0, 7)	0, 53	0 (0, 0)
	Commercial	56	646	1–138	9 (4, 22)	0, 55	0 (0, 2)
	Total	265	1,087	1–138	6 (1, 17)	0, 55	0 (0, 1)
Total		471	1,887	1–138	6 (1, 16)	0, 55	0 (0, 1)

*Q1, 25th percentile; Q3, 75th percentile; Min, minimum; Max, maximum*.

*^a^In all cases, the minimum was 0 and the maximum was 3*.

**Table 3:** Explanatory variables assessed in univariable models for the risk of high farm lung and pleurisy scores in slaughtered finisher pigs from 471 farms in the Philippines.

Explanatory variable	Description	Number of high lung score^§^ farms (%*)	Number of high pleurisy score^§^ farms (%*)	Total No per category
Province	Bulacan	65 (32)	33 (16)	206
	Pampanga	90 (34)	31 (12)	265
Production system	Smallholders	93 (25)	37 (10)	371
	Commercial^†^	62 (62)	27 (27)	100
Farm manager gender	Female	37 (29)	13 (10)	129
	Male	118 (35)	51 (15)	342
Farm manager education	College	91 (42)	36 (16)	219
	Elementary or can’t read	10 (22)	6 (13)	45
	High school	54 (26)	22 (11)	207
Contact with waterfowl, poultry and other animals	No	87 (40)	41 (20)	216
	Yes	68 (27)	23 (9)	255
Contact with neighbors pigs	No	30 (24)	57 (46)	125
	Yes	125 (34)	7(2)	368
Unauthorized entry of people and vehicles	No	16 (31)	4 (8)	52
	Yes	139 (33)	60 (14)	419
Workers living on farm	None	99 (38)	45 (17)	258
	Some or all	56(26)	19 (9)	213
Commercial livestock vehicles allowed on farm	No	35 (23)	6 (4)	149
	Yes	120 (37)	58 (18)	322
Market pen used on farm^†^	No	97 (26)	41 (11)	370
Water source	Yes	58 (57)	23 (23)	101
	Town supply	29 (28)	16 (15)	105
	Other	126 (34)	48 (13)	366
Rats and mice in contact with pigs	No	25 (33)	12 (16)	75
	Yes	130 (33)	52 (13)	396
Prevent disease introduction	Do nothing	97 (40)	42 (17)	245
	Use protective clothing, footbath, downtime, clean vehicles, equipment	58 (26)	22 (10)	226
Clean pens	Other	7 (58)	4 (33)	12
	Daily	148 (32)	60(13)	459
Cleaning products mixed with water	Soap detergent	47 (31)	11 (7)	152
	Disinfectant	108 (34)	53(17)	319
Waste and manure disposal method	Other	124 (29)	48 (11)	426
	Compost or biogas	31(69)	16 (36)	45
Artificial insemination used on farm	No	94 (27)	38 (11)	350
	Yes	61 (50)	26 (21)	121
Introduced or purchased pigs quarantined	No	93 (28)	36 (110)	338
	Yes	62 (47)	28 (21)	133
“Feedback” carried out on this farm^‡^	No	148(33)	57(13)	455
	Yes	7(44)	7(44)	16
Feeding: grower-finishers	Commercial feed	109 (28)	46 (12)	383
	Made on farm feed	46 (52)	18 (20)	88
Swill fed to pigs	No	124 (36)	58 (17)	347
	Yes	31(25)	6 (5)	124
Workers know what swill is	No	66 (33)	35 (17)	202
	Yes	89 (33)	29 (11)	269
Workers can recognize sick and healthy pigs	No	5(45)	1(9)	11
	Yes	150(33)	63(14)	460
Sick pigs separate from healthy pigs	No	94 (27)	38 (11)	350
	Yes	61 (50)	26 (21)	121
Sick and dead pigs recorded	No	105 (27)	39 (10)	389
	Yes	50 (61)	25 (30)	82
Surrounded by piggeries within 500 m	No	78 (29)	36 (13)	271
	Yes	77 (38)	28 (14)	200
*Actinobacillus pleuropneumoniae* like lesion^§^	No	147 (32)	63 (14)	461
	Yes	8 (80)	1 (10)	10
Pericarditis^§^	No	134 (30)	38 (9)	440
	Yes	21 (68)	26 (84)	31
Pleuritis^§^	No	110 (29)	–	380
	Yes	64 (70)		91
Vaccination programs				
*Actinobacillus pleuropneumoniae*	No	118 (29)	48 (12)	409
	Yes	37 (60)	16 (26)	62
Hog cholera	No	53 (36)	18 (12)	146
	Yes	102 (31)	46 (14)	325
*Mycoplasma hyopneumoniae*	No	127 (30)	48 (11)	426
	Yes	28 (62)	16 (36)	45
Porcine circovirus type 2	No	149 (33)	61 (13)	457
	Yes	6 (43)	3 (21)	14
Porcine reproductive and respiratory syndrome	No	109 (29)	45 (12)	377
	Yes	46 (49)	19 (20)	94
Pseudorabies virus	No	121 (29)	49 (12)	422
	Yes	34 (69)	15 (31)	49
Swine influenza virus	No	145 (32)	60 (13)	459
	Yes	10 (83)	4 (33)	12
*Haemophilus parasuis*	No	153 (33)	63 (13)	467
	Yes	2 (50)	1 (25)	4
Atrophic rhinitis	No	153 (33)	64 (14)	469
	Yes	2 (100)	0 (0)	2

*Percentage of total in category. ^†^Market pen is a pig holding area isolated from the main piggery. Pigs that are available for sale are placed in these pens (and not allowed back into the main herd) for livestock traders to view until sold. ^‡^Feeding pig manure, viscera or aborted material to pigs to increase herd immunity. ^§^A farm was classified as being a high lung, pleurisy scores, high pericarditis or high APP-Like lesion farm if at least 50% of pigs from the farm were high lung or pleurisy score pigs, or were classified as high pericarditis or APP-Like lesion, respectively. When a farm had only one pig, the farm was classified based on the lesion status of that pig.

Estimated regression coefficients for the final logistic regression models are shown in Tables 4 and 5. After adjusting for the effect of other covariates in Table 4, commercial farms were 1.85 (95% CI 1.32–2.63; *P* < 0.01) times more likely to be high lung score farms compared with smallholder farms. Among other significant variables, farms that did not vaccinate against hog cholera (RR 1.74, 95% CI 1.28–2.35; *P* < 0.01), had a market pen (RR 1.79, 95% CI 1.18–2.57; *P* < 0.01), or had at least one other pig farm located within 500 m (RR 1.52, 95% CI 1.14–203, *P* < 0.01) were also at higher risk of being high lung score farms.

**Table 4:** Estimated regression coefficients and RRs for the effect of farm-level risk factors on the likelihood of classification of a farm as high lung score farm derived from a multivariable logistic regression model using data from 471 farms collected between October 2011 and March 2012 in the Philippines.

Variable	Coefficients (SE)	RR (95% CI)	P*
Production system			<0.01
Smallholder farms	Reference		
Commercial farms^†^	1.19 (0.33)	1.85 (1.32–2.63)	
Grower-finishers vaccinated against Hog Cholera			<0.01
Yes	Reference		
No	0.88 (0.26)	1.74 (1.28–2.35)	
Market pen used on farm			<0.01
No	Reference		
Yes	0.96 (0.36)	1.79 (1.18–2.57)	
At least one other pig farm within 500 m			0.01
No	Reference		
Yes	0.63 (0.23)	1.52 (1.14–2.03)	
*Actinobacillus pleuropneumoniae* like lesion^‡^			0.01
Absent	Reference		
Present	2.61 (0.86)	146.10 (20.21–830.23)	
High pleuritis score^c^			0.01
No	Reference		
Yes	1.77 (0.32)	22.79 (11.37–44.47)	
Heat producing units			0.98
0.85 or less	Reference		
0.85–1.76	−0.23 (0.33)	0.88 (0.54–1.34)	
>1.76	−0.18 (0.32)	0.89 (0.62–1.34)	

*RR, risk ratio; CI, confidence interval*.

*Intercept coefficient (SE): −1.87 (0.32), *P* < 0.01. Hosmer–Lemeshow Goodness of Fit X^2^_7.944,df 8_; *P* = 0.44*.

*The overall significance of the variable was assessed using a likelihood ratio test.

*^†^Interpretation: after adjusting for the effect of other variables in the model, commercial farms were 1.85 (95% CI 1.32–2.63) times more likely to be classified as high lung score farms compared with smallholder farms*.

*^‡^A farm was classified as being a high lung, pleurisy scores, or high APP-Like lesion farm if at least 50% of pigs from the farm were high lung or pleurisy score pigs, or were classified as high APP-like lesion, respectively. When a farm had only one pig, the farm was classified based on the lesion status of that pig*.

Commercial farms were also at greater risk of being high pleurisy score farms (RR 2.69, 95% CI 1.61–4.42; *P* < 0.01) compared with smallholder farms. Farms that practiced feedback (RR 3.27, 95% CI 1.49–5.63, *P* < 0.01) or allowed commercial livestock vehicles on farm (RR 1.83, 95% CI 1.07–3.20; *P* = 0.03) were at higher risk of being high pleurisy score farms (Table 5).

**Table 5:** Estimated regression coefficients and RRs for the effect of farm-level risk factors on the likelihood of classification of a farm as high pleurisy score farm* derived from a multivariable logistic regression model using data from 471 farms collected between October 2011 and March 2012 in the Philippines.

Variable	Coefficients (SE)	RR (95% CI)	*P*^†^
Production system			<0.01
Smallholder	Reference		
Commercial farms^‡^	1.21 (0.33)	2.69 (1.61–4.42)	
“Feedback” carried out on this farm			0.02
No	Reference		
Yes	1.58 (0.56)	3.27 (1.49–5.63)	
Commercial livestock vehicles allowed on farm			0.03
Yes	Reference		
No	0.69 (0.32)	1.83 (1.07–3.20)	
Heat producing units^§^			0.06
0.85 or less	Reference		
0.85–1.76	−0.31 (0.41)	0.76 (0.38–1.54)	
>1.76	−0.77 (0.40)	0.89 (0.28–1.01)	

*RR, risk ratio; CI, confidence interval. Intercept coefficient (SE): −2.25 (0.34), *P* < 0.01. Hosmer–Lemeshow Goodness of Fit X^2^_3.827,df 8_; *P* = 0.87*.

*High pleuritis score: a farm was classified as being a high pleurisy score farm if at least 50% of pigs from the farm were pleurisy score pigs. When a farm had only one pig, the farm was classified based on the pleurisy status of that pig.

*^†^The overall significance of the variable was assessed using a likelihood ratio test*.

*^‡^Interpretation: after adjusting for the effect of other variables in the model commercial farms were 2.69 (95% CI 1.61–4.42) times more likely to be classified as high pleuritis score farms compared with smallholder farms*.

*^§^Heat production units (HPUs) served as a proxy for herd size and were calculated as described by Zhuang et al. (19)*.

**Figure 1:**
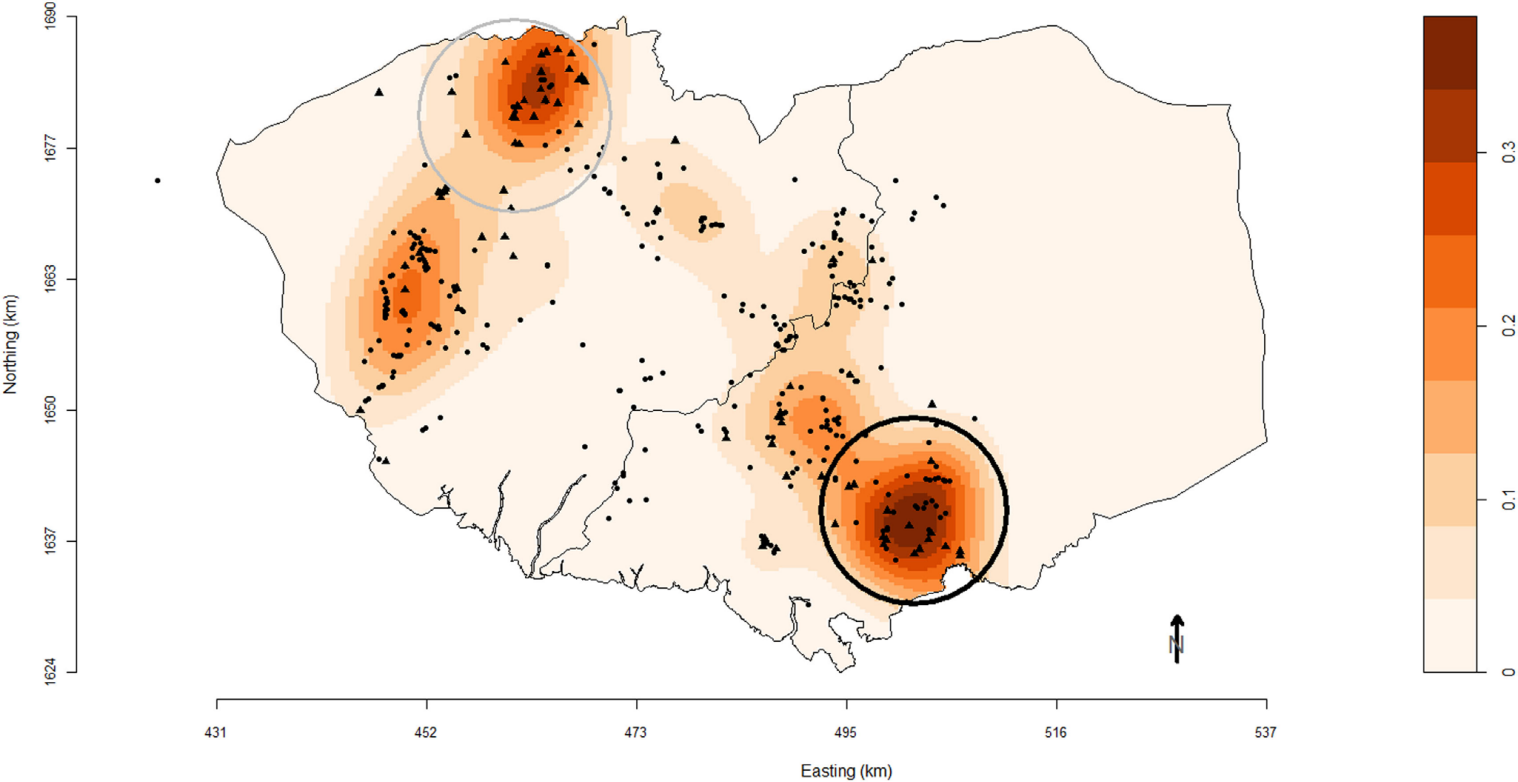
Map of Pampanga (left) and Bulacan (right) provinces showing the location of pig farms (black solid circles, smallholder farms; black solid triangles, commercial farms) participating in the study conducted between October 2011 and March 2012. Farm locations are superimposed over a density plot of high-risk farms as defined in this study. Densities are expressed as the number of respiratory disease high-risk pig farms per square kilometer. Black and gray circles are the primary and secondary spatial clusters of high-risk farms identified using SaTScan™ version 9.1.1 software based on a purely spatial Bernoulli distributional assumption model and scanning for circular clusters with a maximal population threshold of 50% of sites.

One primary spatial cluster was observed in the south-east of Bulacan (Figure 1). The 9.5 km-radius cluster (*P* = 0.01) comprised 39 farms (17 commercial and 22 smallholder farms). Another secondary cluster (*P* = 0.33) was identified in north of Pampanga with a 9.7 km-radius and included 31 farms (22 commercial and 9 smallholder farms).

## Discussion

The prevalence of lung lesions in this study was high, suggesting that a large proportion of animals in Region 3 are likely exposed to a variety of major respiratory pathogens throughout the production cycle. The results are in agreement with prior reports for intensive pig production systems. In South-East Spain, Fraile et al. (25) reported pleurisy and cranioventral pneumonia consolidations in 26.8 and 55.7% of lungs (*n* = 11,000). In Western France, Fablet et al. (7) reported that pneumonia (69.3% of lungs) and pleurisy (15% of lungs) were the most frequent lesions seen at slaughter (using 3,731 lungs from 125 herds), and *M. hyopneumoniae, P. multocida*, and *A. pleuropneumoniae* were the most prevalent pathogens detected within gross lung lesions, 69.3, 36.9, and 20.7% of lungs, respectively. The results from our study indicate that one in two slaughter-age animals were classified with acute lung lesions (51%), and one in four classified with chronic lung lesions (27%). The likelihood of observing lesions at slaughter during a lung scoring study is dependent on time of exposure during the production cycle, number of respiratory pathogens involved, and immune response of animal (5, 25).

Commercial farms were significantly more likely to be high lung score and high pleurisy score farms compared with smallholder farms. Differences in management factors between commercial and smallholder farms such as a higher frequency of purchasing replacement animals and having a continuous production system in commercial farm groups may have contributed to this increased risk. Mixing of animals from different sources of unknown disease status could increase the amount and range of pathogens to which a pig is exposed, posing a greater risk of introducing and maintaining infectious agents in the herd (26). This was attributed to increased social stress which may negatively impact on animals’ immune response and increase their risk of disease (6, 27, 28). Smallholder farmers on the other hand may have accidentally adopted, due to logistic or economic constraints, an all-in-all-out system. An all-in-all-out system has been reported to reduce the risk of introduction of disease to the herd (6, 29).

The presence of another piggery within 500 m (as a proxy for farm density), use of a market pen (a pig holding area isolated from the main piggery where pigs that are available for sale are placed in these pens and not allowed back into the main herd) and adopting appropriate vaccination programs were associated with high farm lung and pleurisy scores. High animal density increases the within-herd prevalence of disease, possibly due to the likelihood that a larger number of animals would be excreting pathogens, thus increasing the load in the environment. As well, the high density of farms increases the risk of between herds transmission of disease either directly through animal-to-animal contact (most likely within smallholder farms) or indirectly through mechanical vectors or iatrogenic routes (30, 31). Improving farm management practices by increasing awareness about possible consequences of feeding practices like feedback use and controlling commercial livestock vehicle access to the farm are risk factors which are manageable. A common awareness that a significant association between farm lung and pleurisy scores and these factors exist would likely result in a marked reduction in prevalence.

The density map of farms with respiratory disease demonstrated the spatial heterogeneity of risk areas in Region 3. Also the use of SatScan™ identified high-risk farm clusters. Identified clusters could be prioritized by regulatory authorities for further investigations or for coordinating intervention efforts. The observed geographical clustering of high-risk farms followed population density and areas where commercial farms are located. A recommendation from this work is that pig farms located at high-risk areas need support and encouragement to adhere to a high standard of biosecurity measures to prevent or slow down the risk of disease introduction in their herds.

Tracing back slaughtered pigs to their source farms through information collected from livestock traders at slaughterhouses in Region 3 demonstrated that an effective trace-back system is achievable in Region 3. This study identified pig movements from other provinces into Region 3 that involved pigs with different disease pattern compared with those in Region 3. This information is useful for local veterinary authorities and allows them to adopt strategies that enforce disease control activities with greater precision. In the absence of traceability system or up-to-date farm data, knowledge of the characteristics of pigs (or source farms) that render them more likely to be atypical would be of value, since this information could be used to inform a risk-based approach to disease surveillance and control.

There are some limitations with the design of the current study. The desired sample size (640 pig farms were required for this study) was not achieved due to the inconsistencies in the number of farms that send their pigs to slaughter during the study period. The lower sample size could influence the external validity of the current study. Our results represent only the slaughtered population of farmed pigs, specifically those with no clinical or subclinical signs of disease, as animals presented at abattoirs must pass an antemortem veterinary inspection to be eligible for slaughter for human consumption. Sampling at abattoirs is thus recognized to have a bias analogous to the “healthy worker effect” in human occupational studies (32), i.e., the working (or abattoir) population is likely to be healthier than the general population. Hence, the abattoir population is likely to be healthier than the general population. In this case, the direction of this bias is likely to be toward the null, therefore underestimating the strength of association between the observed risk factors and the outcome. However, this study aimed to report prevalence of lung lesions in pigs submitted for slaughter (i.e., in clinically and subclinically diseased pigs), and the bias described is unlikely to have a large effect on the population estimates reported. Misclassification bias in this study was unlikely. Pigs, and therefore farms, were classified as having low or high and pleuritis scores based on independent lung scoring conducted by one experienced veterinarian at the time of slaughter. Also classifying pigs based on their lung and pleuritis scores is independent of the exposure variables rendering misclassification bias unlikely. Finally, it is worth noting that the final statistical analyses were at the farm level. Therefore, our findings that exposure to the risk factors increase the risk of high lung scores and high pleurisy scores at the farm level does not necessarily mean that exposure to these factors increases the risk at the pig level. This bias is likely to be away from the null, therefore exaggerating the effects of exposure on the risk of disease at the farm level (20).

In conclusion, commercial farms were at higher risk than smallholder farms of being classified as high lung and pleurisy score farms. Presence of a market pen on farm, not vaccinating grower-finisher pigs against Hog Cholera and the presence of another piggery within 500 m were also associated with the risk of a farm being a high lung score farm. Practicing feedback and allowing commercial livestock vehicles on farm were associated with the risk of a farm being a high pleurisy score farm. Farmer should be encouraged and supported to review and improve on-farm disease control programs to reduce risk of high lung and pleurisy scores in their herds. Not only does this study inform pig producers in the Philippines about potential factors influencing the health and productivity of their herds, it also provides valuable insights for veterinary authorities to evaluate the feasibility of implementing routine inspection of pig lungs at slaughter to monitor herd health in the Philippines.

## Ethics Statement

All data collection for this study was conducted in accordance with the accepted guidelines of the Livestock Research Division, Philippines, and was approved by Officer-in-charge, Animal Health Division, Philippines. Not all farmers were literate enough to understand a written consent form, so to be consistent, it was decided to obtain a verbal consent from all participants involved in the survey. Verbal consent was noted on the questionnaire used for the farmer interview. The survey responses used in this study were anonymized by the survey team, who are the co-authors of this scientific paper. Because all activities were carried out as part of routine veterinary surveillance and disease investigation activities by the Department of Agriculture Region 3, no additional animal or human ethics approval was required for this research. Interviews and sample collection were performed in accordance with the relevant guidelines and regulations of the Department of Agriculture Services, Region 3. The Department of Agriculture Services, Region 3 could respond to specific questions on the conduct of the farmer survey if such questions arise. All lung scores were conducted at the participating slaughterhouses by an experienced veterinarian from the Department of Agriculture Services, Region 3 using standardized lung scoring methods. No pigs were slaughtered for the purpose of this study.

## Author Contributions

JA was responsible for drafting the manuscript, study design, and data analyses. CP, TB, JM, and PB assisted with drafting the manuscript and study design. EV, EL, and JD assisted with the study design and conducting field activities. ML, VB, and AB also assisted in the study design and overseeing field activities.

## Conflict of Interest Statement

The authors declare that the research was conducted in the absence of any commercial or financial relationships that could be construed as a potential conflict of interest.
